# Deltex 2 Mediates Oxidative Stress and Neurotoxicity in Freezing of Gait of Parkinson's Disease via the Notch2‐Nrf2 Axis

**DOI:** 10.1002/cns.71135

**Published:** 2026-09-18

**Authors:** Qibin Zheng, Xiaohua Zheng, Junxin Zhao, Lin Lin, Yibiao Chen, Huiqing Wang, Zhangya Lin, Lianghong Yu

**Affiliations:** ^1^ Department of Neurosurgery, National Regional Medical Center, the First Affiliated Hospital, Binhai Campus of the First Affiliated Hospital Fujian Medical University Fuzhou China; ^2^ Fujian Provincial Institutes of Brain Disorders and Brain Sciences, the First Affiliated Hospital Fujian Medical University Fuzhou China; ^3^ Fujian Provincial Clinical Research Center for Neurological Diseases, the First Affiliated Hospital Fujian Medical University Fuzhou China; ^4^ Pain Research Institute, Fujian Provincial Key Laboratory of Brain Aging and Neurodegenerative Diseases, School of Basic Medical Sciences Fujian Medical University Fuzhou China; ^5^ Fujian Maternity and Child Health Hospital, College of Clinical Medicine for Obstetrics & Gynecology and Pediatrics Fujian Medical University Fuzhou China; ^6^ Department of Neurology The Second Affiliated Hospital of Fujian Medical University Quanzhou China

**Keywords:** dopamine, freezing of gait, oxidative stress, Parkinson's disease, tyrosine hydroxylase

## Abstract

**Background:**

Freezing of gait (FOG) is a disabling symptom of advanced Parkinson's disease (PD) with limited therapeutic options. This study identifies Deltex 2 (DTX2) as a novel molecular driver of FOG and elucidates its mechanism.

**Methods:**

FOG rat models were established by bilateral injections of 6‐hydroxydopamine (6‐OHDA) into the dorsal striatum and bilateral infusions of immunotoxin 192 IgG‐saporin into the basal forebrain regions. Proteomic sequencing was performed to identify differentially expressed proteins in the striatum. In vitro studies employed human dopaminergic‐like SH‐SY5Y cells differentiated with retinoic acid. Cellular responses were evaluated by assessing cell viability, apoptosis, reactive oxygen species (ROS) levels, dopamine content, and tyrosine hydroxylase (TH) expression.

**Results:**

Proteomic profiling revealed significantly elevated DTX2 levels in the striatum of FOG model rats. In vitro, DTX2 knockdown in differentiated SH‐SY5Y cells conferred robust protection against 6‐OHDA‐induced neurotoxicity and oxidative stress, as evidenced by increased cell viability, reduced apoptosis, and diminished ROS accumulation. DTX2 silencing restored dopamine synthesis and TH expression. Mechanistically, DTX2 promoted oxidative stress by ubiquitinating and degrading Notch2, a positive regulator of Nrf2 transcriptional activity. Conversely, DTX2 interference stabilized Notch2, thereby enhancing Nrf2 signaling and antioxidant defense. In vivo, striatal knockdown of DTX2 in FOG rats markedly ameliorated gait abnormalities and improved survival of dopaminergic neurons.

**Conclusion:**

DTX2 emerges as a key molecular driver of freezing of gait in PD. Pharmacological or genetic suppression of DTX2 stabilizes the Notch2–Nrf2 antioxidant axis, rescuing neurons and restoring motor performance in rodent models. These findings identify DTX2 as a readily druggable target for precision therapy aimed at alleviating FOG and improving mobility in patients with advanced PD.

## Graphical abstract

Under normal conditions, Notch2 promotes Nrf2 transcription by forming a transcriptional activation complex, which is crucial for maintaining the antioxidant capacity of human dopamine‐like neural cells. In pathological conditions, increased DTX2 expression has been shown to mediate Notch2 ubiquitination and subsequent degradation, leading to reduced Nrf2 transcription. Consequently, this results in elevated cellular oxidative stress, causing impairment of cellular viability and function, and thereby promoting FOG progression.

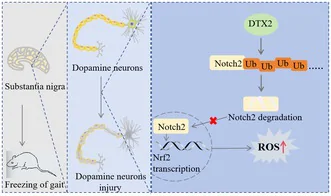

## 1 Introduction

Parkinson's disease (PD) is a chronic neurodegenerative disorder that is characterized primarily by resting tremor, muscle rigidity, and bradykinesia. Postural instability and gait disturbances frequently emerge in the mid‐to‐late stage of the condition [1]. Postural instability primarily manifests as trunk tilt, spinal deformity, and impaired body stability [2]. Gait disturbances primarily encompass shuffling gait, freezing of gait (FOG), and other presentations such as altered stride pattern, reduced step length, disrupted walking rhythm, and impaired dual‐task performance [3]. FOG is characterized as a transient, episodic disability that impedes effective ambulation under the intention to walk. FOG has been observed in up to 90% of patients with Hoehn and Yahr Stage 4 PD, thus serving as an indicator of PD progression into the mid‐to‐late stages [4]. FOG precipitates a cascade of adverse events—falls, injuries, and fragility fractures—that exponentially amplify the disease burden and catastrophically erode quality of life. Although clinicians can deploy pharmacotherapy, deep‐brain stimulation, physiotherapy, and multidisciplinary rehabilitation, these tools provide only symptomatic relief. The pathogenesis of FOG remains enigmatic, and no evidence‐based pharmacological intervention has yet been approved to prevent or abort this paroxysmal immobility in Parkinson's disease.

Dopaminergic neurons of the substantia nigra pars compacta (SNc) constitute a vital midbrain population whose axons converge on the striatum (caudate and putamen), forging the nigrostriatal pathway. By releasing dopamine, these cells tune basal‐ganglia circuitry that governs voluntary movement, reward processing, and executive cognition [5]. Dopamine has been demonstrated to facilitate the direct pathway, thereby promoting movement, and to impede the indirect pathway, thus reducing movement inhibition. This effect is achieved by activating D1 and D2 receptors in the striatum, thus coordinating the initiation and smoothness of movement [6]. Degeneration of dopaminergic neurons is a hallmark of the pathological features of PD, resulting in symptoms such as FOG [7]. However, the molecular mechanism of FOG remains to be elucidated.

In PD, the progressive degeneration of dopaminergic neurons is primarily caused by neurotoxic injury. Extensive research indicates that oxidative stress can induce neurotoxic injury through multiple pathways, serving as a key pathological mechanism in various neurodegenerative diseases, including PD [8, 9, 10]. Excessive production of reactive oxygen species (ROS) and inadequate antioxidant mechanisms can result in oxidative stress, which in turn can damage various components of nerve cells, including lipids, proteins, and DNA. In the brains of patients with PD, there is a significant elevation in oxidative stress levels, which causes widespread damage to neurons and further exacerbates gait control disorders [9]. Oxidative stress has been demonstrated to damage neurons in the basal ganglia, particularly dopaminergic neurons involved in motor control. This damage can result in impaired motor signal transmission and an elevated risk of FOG [11]. Consequently, elucidating the oxidative stress mechanisms in dopaminergic neurons holds promise for improving FOG symptoms and enhancing the quality of life for PD patients by reducing neurotoxic injury.

In this study, we established a FOG rat model and performed quantitative proteomics of striatal tissue to identify differentially expressed proteins. We hypothesized that dysregulated protein degradation mechanisms contribute to FOG pathogenesis and investigated the potential role of the E3 ubiquitin ligase Deltex‐2 (DTX2) in dopaminergic neurodegeneration. This work aims to elucidate novel molecular targets for FOG intervention in Parkinson's disease.

## 2 Materials and Methods

### 2.1 Rat Grouping

The objective of this study was to investigate the gait and balance impairments associated with PD‐related falls. To this end, rats with dual cholinergic‐dopamine losses were utilized as subjects [12]. A total of 30 male Sprague Dawley rats (Cavens Experimental Animal Co. Ltd., Changzhou, Jiangsu, China) were utilized in this study, with 10 rats allocated to the sham group and 20 rats assigned to the FOG group. Only male rats were used in this study because PD shows a higher incidence in men [13, 14]. Age‐matched male and female SD rats differ markedly in body weight, which influences behavioral readouts; consequently, we followed published precedent and selected males [15, 16].

### 2.2 Induction of the FOG Rat Model

FOG‐group rats (*n* = 20) were anesthetized with isoflurane (SurgiVet Isotec 4): induction at 4%–5%, maintenance at 2%–3% in 0.6 L/min O_2_. Animals were positioned in a stereotaxic frame, core temperature held at 37°C with a servo‐controlled heating pad, and eyes protected with ophthalmic ointment. Prophylactic 0.9% NaCl (1 mL/100 g, *s.c*.) prevented hypovolemia, and desipramine hydrochloride (10 mg/kg, *i.p*.) was given to safeguard noradrenergic neurons prior to neurotoxin delivery.

The rats underwent bilateral injections of 6‐hydroxydopamine (6‐OHDA) into the dorsal striatum and bilateral infusions of immunotoxin 192 IgG‐saporin into the basal forebrain regions. During the 6‐OHDA injections, 6.0 μg/2 μL of 6‐OHDA was prepared in a solution of 0.9% NaCl and 0.1% ascorbic acid. Four injection sites were targeted in the dorsal striatum, with two sites per hemisphere: (i) left hemisphere: anterior to bregma: 1.2 mm, lateral to midline: −2.5 mm, ventral to skull surface: 4.8 mm (Site left 1); anterior to bregma: 0.2 mm, lateral to midline: −3.0 mm, ventral to skull surface: 5.0 mm (Site left 2). (ii) Right hemisphere: anterior to bregma: 1.2 mm, lateral to midline: +2.5 mm, ventral to skull surface: 4.8 mm (Site right 1); anterior to bregma: 0.2 mm, lateral to midline: +3.0 mm, ventral to skull surface: 5.0 mm (Site right 2). During the immunotoxin 192 IgG‐saporin infusions, 120 ng/μL of immunotoxin 192 IgG‐saporin was dissolved in artificial cerebrospinal fluid (aCSF), with 0.5 μL infused per hemisphere. Two injection sites were targeted in the basal forebrain: (i) Left hemisphere: posterior to bregma: −0.8 mm, lateral to midline: −2.9 mm, ventral to skull surface: 7.8 mm. (ii) Right hemisphere: posterior to bregma: −0.8 mm, lateral to midline: +2.9 mm, ventral to skull surface: 7.8 mm.

The coordinates were marked, and a 0.4 mm drill bit was used with a high‐speed dental drill to penetrate the skull to the dura mater. The edges of the bone window exhibited signs of slight abrasion, resulting in a diameter of 0.6–0.8 mm. The injection was carried out at a rate of 1 μL/min. Subsequent to the injection, the needle was maintained in position for a period of 5–8 min to facilitate diffusion. Subsequently, the needle was retracted at a rate of 1 mm/min. The incision was closed with non‐absorbable nylon sutures, and a topical antibiotic was applied to the wound.

Two weeks before surgery, every rat—sham and FOG—was habituated to the CatWalk runway. After a 5‐week recovery, gait was recorded; animals were then euthanized. Striatal and cortical tissue from CatWalk‐tested rats was snap‐frozen for sequencing, whereas brains from non‐tested cohorts were reserved for immunohistochemistry.

### 2.3 Catwalk Runway Training

Prior to the surgical procedures, a one‐week adaptation training period was implemented to acclimate the rats to the Catwalk gait analysis system and ensure accurate data collection post‐surgery. The objective of the training was to acclimate the rats to the runway environment without the collection of any data, thereby establishing a stable reward mechanism and controlling body weight. Prior to the initiation of the training, the rats were subjected to a period of food deprivation, a method employed to enhance motivation, and water was provided ad libitum. Subsequent to the conclusion of the training session, each rat was provided with 12–15 g of food to reinforce the training and maintain body weight. The Catwalk system and all related equipment were activated, and the laboratory lights were deactivated to establish a dim, controlled environment conducive to the rats' movement. Each rat was meticulously positioned at the origin of the Catwalk runway and permitted to traverse the runway autonomously, without any external stimuli. The experimental subjects, henceforth designated “rats,” underwent training on a daily basis, with the objective of completing three to five uninterrupted, continuous traversals of the designated runway. The training criterion for success was defined as the rat's ability to traverse the runway three times without interruption, with each traversal consisting of at least 10 steps. The adaptation training program was conducted over the course of 1 week, involving daily sessions to acclimate the rats to the runway environment. Subsequent to the adaptation training, the Catwalk XT system (Noldus Information Technology, Wageningen, the Netherlands) settings were adjusted based on the rats' performance to optimize data collection. The experimental configuration encompassed various settings, including background light intensity, camera sensitivity, and the total duration for data inclusion. The optimized settings were then maintained constant throughout the experiment to ensure consistency in data collection. Subsequent to the 1‐week adaptation training, baseline Catwalk data were collected over a period of three consecutive days. This baseline data served as a reference point for comparing post‐surgical gait changes.

Five weeks post‐surgery, FOG severity was gauged by three CatWalk trials: rats completing every run were rated mild; any rat that failed even one trial was rated severe. In the FOG group, the probability of being unable to complete the test due to severe FOG episodes (starting hesitation or mid‐run freezing) was 34/60 (56.67%). Among them, rats that completed all 3 runway tests were considered mild, totaling 8 rats; rats with FOG episodes were considered severe, totaling 12 rats, of which 10 failed to obtain any data, and 2 obtained data from one test. Subsequently, 5 rats from the Sham, mild, and severe groups were randomly selected for evaluation and detection using the Catwalk gait analysis.

### 2.4 Catwalk Gait Analysis

After the runway tests, five rats were randomly selected from each of the sham, mild, and severe groups for detailed gait analysis with the CatWalk XT system (Noldus Information Technology, Wageningen, the Netherlands). The system records footprints as rats cross a glass plate illuminated from below, enabling measurement of stride length, step width, and foot placement. Each rat was allowed multiple crossings; data were processed with CatWalk software and compared across groups to identify differences linked to lesion severity. Among the severe group, 2 rats had severe starting hesitation and failed to obtain data, leaving only 3 rats that completed the assessment. Therefore, the CatWalk analysis data for the severe group included only 3 rats, but the subsequent sequencing samples had *n* = 5 for each group.

### 2.5 Perfusion and Brain Harvesting

The rats were anesthetized with an intraperitoneal injection of 3% pentobarbital sodium (0.15 mL/100 g) to prepare brain tissues for histological analysis. Subsequently, the rats were positioned in a supine stance. The abdominal cavity was opened transversely below the xiphoid process to expose the liver and xiphoid. The xiphoid was meticulously lifted using a hemostat, thereby exposing the diaphragm, which was subsequently punctured. The ribs were then meticulously removed along the anterior axillary lines on both sides of the rat to create a thorough opening of the thoracic cavity. The heart was exposed through a blunt dissection technique, utilizing a vascular clamp for precise control. A perfusion needle was inserted into the left ventricle through the apex of the heart and advanced into the aorta. Subsequent to the injection of fluid into the aorta, the needle was secured with a hemostat, and the right atrium was opened to permit the egress of dark venous blood. The perfusion process was initiated with 300 mL of saline at a low flow rate, followed by 300 mL of 4% paraformaldehyde at 4 degrees Celsius. The initial acceleration of the perfusion was followed by a subsequent deceleration after the administration of 150 mL, with the objective of mitigating potential tissue damage. The efficacy of the perfusion process was determined by the color change of the liver, which transitioned from red to yellow and ultimately to white. This chromatic shift was accompanied by the efflux of a clear liquid from either the right atrium or the distal tip of the tail (or nose). Subsequent to perfusion, the rat was decapitated, the skull was extracted, and the brain was meticulously extracted and decontaminated of meninges. Subsequently, the brain was immersed in 4% paraformaldehyde for a period of 48 h, thereby facilitating fixation. For the purpose of studying dehydration, the brain was transferred to a sucrose solution composed of 25% sucrose. This process was repeated until the brain was completely submerged in the solution. Subsequently, the brain was transferred to a sucrose solution composed of 30% sucrose, and the process was repeated until the brain sank in the solution once more. Subsequent to the cleaning of the surface solution, the brain was placed in a −80°C freezer for a period of 1 h for cryosectioning. Alternatively, the sample was stored at −80°C for an extended period, with the intention of future use.

### 2.6 Immunohistochemical Staining of Brain

A comprehensive evaluation of neuronal loss in various brain regions was conducted, with a particular focus on the analysis of dopaminergic neurons in the caudate putamen (CPu) among the sham, mild, and severe groups. The implementation of immunohistochemical staining techniques enabled the analysis in question [17]. Serial coronal sections (40‐μm thick) were obtained using a cryostat and collected in six‐well plates filled with antifreeze solution (30% ethylene glycol, 30% glycerol, and 0.1 M phosphate buffer). To detect dopaminergic neurons in the caudate putamen (CPu), sections were subjected to immunohistochemical staining using tyrosine hydroxylase (TH) as a marker. Free‐floating sections were initially washed thrice in 0.01 M phosphate‐buffered saline (PBS) for a duration of 10 min each. The endogenous peroxidase activity was quenched by incubating the sections in 0.3% hydrogen peroxide in PBS for a period of 30 min. Subsequent to this, the sections were washed again in PBS and blocked with 10% normal goat serum (NGS) in PBS for 1 h at room temperature to prevent nonspecific binding. Subsequently, the sections were subjected to an overnight incubation at 4°C with the primary antibody against TH (1:500; ab137869, Abcam, Cambridge, UK), which had been diluted in PBS containing 0.3% Triton X‐100 and 1% NGS. Following a thorough washing step with PBS, the sections were subjected to an incubation with a biotin‐conjugated goat anti‐rabbit secondary antibody for a duration of 2 h at room temperature. Following an additional series of washes in PBS, the sections were subjected to an incubation period of 1 h at room temperature with the avidin‐biotin peroxidase complex, in accordance with the manufacturer's guidelines. The immunoreaction was subsequently subjected to visualization through incubation with 0.05% 3,3′‐diaminobenzidine (DAB) and 0.01% hydrogen peroxide for approximately 5 min. Subsequently, the sections were washed in PBS, mounted onto gelatin‐coated slides, air‐dried, and coverslipped with Permount.

### 2.7 Proteomic Sequencing and Analysis

For proteomic sequencing, 20 mg of STR and cortex from each group of rats were collected, swiftly frozen in liquid nitrogen, and stored at −80°C (*n* = 5 in each group). To facilitate a comprehensive analysis, the study design incorporated multiple comparison groups, allowing for systematic investigation of the variation in protein expression profiles across these groups. The identification of differentially expressed proteins was achieved by establishing a False Discovery Rate (FDR, also known as adj.*p*.value) threshold of 0.05. This methodological framework facilitated the identification of proteins exhibiting substantial expression disparities among the distinct groups. Conventional pairwise comparisons were supplemented by linear regression analysis within the STR samples to ascertain proteins whose abundance exhibited a linear relationship with the progression of FOG. The progression stages (sham, mild, severe) were coded as numerical parameters in the linear regression model (Stage: 0 for sham, 1 for mild, and 2 for severe). Proteins exhibiting a unidirectional linear trend (either upregulated or downregulated) with an FDR of less than 0.05 were considered significant. This methodological approach facilitated the identification of biomarkers that directly correlate with disease progression.

### 2.8 Induction of Human Dopamine‐Like Neural Cells

The induction of human dopamine‐like neural cells was achieved through the utilization of SH‐SY5Y cells, a type of human neuroblastoma cell line, as previously documented in the literature [18]. These cells were derived from Procell Technology (Wuhan, Hubei, China). To induce differentiation, SH‐SY5Y cells were initially plated at a density of 1 × 10^5^ cells/mL in complete growth medium. Following a 24‐h incubation period, the cells entered the first stage of differentiation, during which they were cultured in a medium containing 3% serum supplemented solely with retinoic acid. The subsequent alterations in cellular morphology, biochemical properties, and gene expression were meticulously evaluated on the seventh day of the differentiation process.

### 2.9 Quantitative Real‐Time PCR (qPCR)

Total RNA was extracted from differentiated SH‐SY5Y cells using the QIAGEN RNeasy Mini Kit (Qiagen, Manchester, UK). The quality and quantity of RNA were assessed using a NanoDrop spectrophotometer. Subsequently, cDNA was synthesized from 1 μg of RNA using the qPCRBIO SyGreen 1‐Step Detect Low‐ROX kit (PCR Biosystems Inc., Wayne, Pennsylvania, USA). qPCR was then performed using gene‐specific primers for DTX2, Notch2, Nrf2, and GAPDH, which served as a housekeeping gene. The reaction mixture was subjected to 40 cycles of amplification (95°C for 5 s, 60°C for 30 s) on a Bio‐Rad iQ5 Optical Module PCR Detection System (Bio‐Rad, Hercules, CA, USA). The relative expression levels of DTX2, Notch2, and Nrf2 mRNA were subsequently calculated using the 2^−ΔΔCt^ method. Primers were listed in Table 1.

**TABLE 1:** Primer sequences used in this study.

Gene	Forward	Reverse
hGAPDH	AGGGCTGCTTTTAACTCTGGT	CCCCACTTGATTTTGGAGGGA
hDTX2	GCCAGTGCTACCTTCCAGAC	GCGGTCCATCTCTGTCTTGT
hNotch2	CTTCAGTGGTATGGACTGTGAG	GCAGAGGCAGGAGAAAGTATT
hNrf2	CACATCCAGTCAGAAACCAGTGG	GGAATGTCTGCGCCAAAAGCTG

### 2.10 Western Blot Analysis

Protein samples were extracted from differentiated SH‐SY5Y cells using a lysis buffer containing protease inhibitors to ensure protein integrity. The protein concentration was subsequently quantified using a BCA protein assay kit (Beyotime, Beijing, China). Equal amounts of protein were resolved by sodium dodecyl sulfate‐polyacrylamide gel electrophoresis (SDS‐PAGE) and transferred onto polyvinylidene difluoride (PVDF) membranes. The membranes were blocked with 5% skim milk in TRIS‐buffered saline with 0.5% Tween 20 (TBST) for 1 h at room temperature to prevent nonspecific binding. Subsequently, the membranes were incubated with primary antibodies against DTX2 (1:500; PA5‐100200; Invitrogen, Waltham, MA, USA), Bax (1:1000; MA5‐35342; Invitrogen, Waltham, MA, USA), Bcl2 (1:500; PA5‐27094; Invitrogen, Waltham, MA, USA), Caspase 3 (1:500; PA5‐77887; Invitrogen, Waltham, MA, USA), cleaved Caspase 3 (1:500; MA5‐35335; Invitrogen, Waltham, MA, USA), SOD1 (1 μg/mL; PA1‐30195; Invitrogen, Waltham, MA, USA), SOD2 (1:500; PA5‐30604; Invitrogen, Waltham, MA, USA), Catalase (1:500; PA5‐29183; Invitrogen, Waltham, MA, USA), TH (1:1000; OPA1‐04050; Invitrogen, Waltham, MA, USA), Nr3c1 (4 μg/mL; PA1‐516; Invitrogen, Waltham, MA, USA), Notch2 (0.01 μg/mL; PA5149980; Invitrogen, Waltham, MA, USA), Nrf2 (1:500; PA5‐27882; Invitrogen, Waltham, MA, USA), and β‐actin (1:500; PA1‐183; Invitrogen, Waltham, MA, USA) overnight at 4°C. Following a thorough washing step with TBST, the membranes were then subjected to an incubation with horseradish peroxidase‐conjugated secondary antibodies for a duration of 1 h at ambient temperature. The visualization of protein bands was accomplished through the utilization of a chemiluminescent detection system.

### 2.11 siRNA Transfection and 6‐OHDA‐Induced Neurotoxicity in Differentiated SH‐SY5Y Cells

In this study, a meticulous approach was adopted to examine the impact of small interfering RNA (siRNA) on the differentiation of SH‐SY5Y cells. The siRNAs utilized in this study targeted DTX2 (siDTX2), Notch2 (siNotch2), or a negative control siNC. The siRNAs were procured from RiboBio Technology (Guangzhou, Guangdong, China), and the Lipofectamine RNAiMAX reagent was obtained from Thermo Fisher Scientific (Waltham, MA, USA). The cells were then subjected to differentiation, a process that was carried out in strict accordance with the manufacturer's instructions for the use of the Lipofectamine RNAiMAX reagent. The transfection efficiency was verified 48 h post‐transfection. Subsequently, the cells were exposed to 10 μg/mL 6‐OHDA for a period of 24 h in order to induce neurotoxicity [19]. The assessment of cell viability was conducted by employing the MTT assay [20]. The expression levels of DTX2 and Notch2, along with their associated proteins, were subjected to analysis through qPCR and Western blot analysis.

### 2.12 Plasmid Transfection and Treatment of SH‐SY5Y Cells

Differentiated SH‐SY5Y cells were subjected to transfection using a plasmid expressing DTX2 (OE‐DTX2) or a control plasmid (OE‐NC) (RiboBio Technology, Guangzhou, Guangdong, China). Following transfection, the cells were subjected to a 24‐h treatment with 10 mM N‐acetyl cysteine (NAC), a ROS inhibitor. The assessment of cell viability was conducted by means of the MTT assay. The Notch2 overexpressing plasmid (OE‐Notch2) from RiboBio Technology (Guangzhou, Guangdong, China) was utilized to overexpress Notch2 in differentiated SH‐SY5Y cells.

### 2.13 Annexin V/PI Apoptosis Assay

Differentiated SH‐SY5Y cells that had been exposed to 6‐OHDA/NAC were harvested and washed with cold PBS. The cells were resuspended in binding buffer and stained with Annexin V‐FITC and PI according to the manufacturer's protocol (Beyotime, Beijing, China). The stained cells were subsequently analyzed by flow cytometry (FACSCanto II; BD Biosciences, San Jose, CA, USA) to determine the percentage of apoptotic cells. The subsequent analysis of the data was conducted using the FlowJo software.

### 2.14 Intracellular ROS Detection by DCFH‐DA Staining

In order to assess the intracellular ROS levels in differentiated SH‐SY5Y cells treated with 6‐OHDA/NAC, the cells were stained with 2′,7′‐dichlorofluorescein diacetate (DCFH‐DA) [21]. Following 6‐OHDA treatment, cells were exposed to 10 μM DCFH‐DA at 37°C for 30 min in a dark environment. Subsequent to the incubation period, the cells were washed with PBS to ensure the removal of excess dye. The intensity of the emitted fluorescence was measured in a variety of ways. Initially, the excitation/emission wavelengths of 488/525 nm were utilized to assess the intensity of the fluorescence employing a fluorescence microscope. Subsequently, the intensity of the fluorescence was measured using flow cytometry. The relative fluorescence intensity was employed as a metric to quantify the levels of ROS.

### 2.15 Detection of SOD Activity

The activity of superoxide dismutase (SOD) in differentiated SH‐SY5Y cells treated with 6‐OHDA/NAC was measured using a colorimetric SOD activity assay kit (EIASODC; Invitrogen, Waltham, MA, USA). The SOD activity assay was performed in accordance with the manufacturer's instructions. The degree of absorption was subsequently quantified at a wavelength of 560 nm by employing a microplate reader.

### 2.16 Detection of Dopamine Levels

Dopamine in 6‐OHDA/NAC‐treated SH‐SY5Y lysates was quantified with the KA3838 ELISA kit (Abnova, Taipei, Taiwan) following the manufacturer's protocol. Absorbance was read at 450 nm on a microplate reader, and dopamine concentrations were calculated from the standard curve.

### 2.17 Detection of TH Levels

The levels of TH in the cell lysates of differentiated SH‐SY5Y cells treated with 6‐OHDA/NAC were measured using the GENLISA Human Tyrosine Hydroxylase ELISA Kit (KBH3881; Krishgen Biosystems, Cerritos, CA, USA). The ELISA was performed in strict accordance with the manufacturer's guidelines. The degree of absorption was subsequently quantified at a wavelength of 450 nm by means of a microplate reader.

### 2.18 Immunofluorescence Staining

Differentiated SH‐SY5Y cells that had been treated with 6‐OHDA/NAC were fixed with 4% paraformaldehyde for a period of 15 min at room temperature. Subsequently, permeabilization was induced with 0.1% Triton X‐100 in PBS for a duration of 10 min. Subsequently, the cells were blocked with 5% bovine serum albumin (BSA) in PBS for a duration of 1 h at room temperature, with the objective of minimizing nonspecific binding. Subsequently, cells were incubated with a primary antibody against TH (1:1000; MA5‐47435; Invitrogen, Waltham, MA, USA) overnight at 4°C. Following a thorough washing step with PBS, the cells were subjected to an incubation with a secondary antibody conjugated to Alexa Fluor 488 for a duration of 1 h at ambient temperature. The nuclei were counterstained with DAPI. The visualization of fluorescence was accomplished through the utilization of a fluorescence microscope.

### 2.19 AAV Transfection of FOG Rats

In the FOG rat model, 3 weeks after the 6‐OHDA injection, rats underwent stereotactic injection of adeno‐associated virus AAV‐DTX2‐shRNA (DTX2‐shRNA) and negative control virus (NC‐shRNA) into the striatum and substantia nigra. The injection volume was 2 μL per site at a rate of 0.5 μL per minute, with a total volume of 4 μL per subject. Subsequent determination of the viral titer yielded a result of 1 × 10^13^ vg/mL. Prior to undergoing surgical procedures, all rats received an intraperitoneal injection of 20% d‐mannitol, a solution that has been demonstrated to enhance viral distribution. Each group contained five rats. Two weeks after the viral injection, the rats were subjected to Catwalk gait analysis. Subsequent to the culmination of the Catwalk experiments, the rats were euthanized, and the brain tissues were harvested for further analysis.

### 2.20 TUNEL Staining

Apoptosis in the rat substantia nigra pars compacta (SNpc) was evaluated by TUNEL staining (C10618, Invitrogen, Waltham, MA, USA). Paraffin sections containing the SNpc were deparaffinized, rehydrated, and permeabilized with proteinase K, then incubated with the TUNEL reaction mix per the manufacturer's protocol. Nuclei were counterstained with DAPI.

### 2.21 Chromatin Immunoprecipitation (ChIP) Assay

ChIP was performed to investigate the binding of Notch2 and Rbpj to the Nrf2 promoter region in differentiated SH‐SY5Y cells. The cells were fixed with 1% formaldehyde for 10 min at room temperature to cross‐link proteins to DNA. The cross‐linking reaction was terminated by the addition of glycine to a final concentration of 0.125 M. Subsequently, the cells were lysed, and the nuclei were isolated and sonicated to shear the chromatin into fragments of approximately 200–500 bp. Subsequently, the chromatin lysates were subjected to an overnight incubation at 4°C with antibodies directed against Notch2 (PA5‐27458; Invitrogen, Waltham, MA, USA) and Rbpj (720219; Thermo Fisher Scientific, Waltham, MA, USA). Subsequently, Protein A/G magnetic beads were added to the lysates with the objective of capturing the antibody‐chromatin complexes. Subsequent to the washing and elution of the complexes, the cross‐links were reversed through incubation at 65°C for a duration of one night. The purification of DNA was carried out using a ChIP DNA purification kit (Proteintech, Rosemont, IL, USA). Subsequently, the purified DNA was subjected to qPCR analysis, employing primers that were designed to specifically target the Nrf2 promoter region. The relative enrichment of the Nrf2 promoter was subsequently calculated by comparing the Ct values of the immunoprecipitated samples to those of the input chromatin.

### 2.22 Dual‐Luciferase Reporter Assay

Nrf2 transcriptional activity after Notch2 knockdown was measured with a dual‐luciferase reporter assay. Differentiated SH‐SY5Y cells were co‐transfected with an ARE‐driven firefly luciferase plasmid, Renilla luciferase control, and either siNotch2 or siNC using Lipofectamine 3000 (Thermo Fisher). After 48 h, luciferase activities were quantified with the Dual‐Luciferase kit (DL101‐01, Vazyme) and firefly values were normalized to Renilla. Activity in siNotch2 cells is expressed relative to siNC controls.

### 2.23 Co‐Immunoprecipitation (Co‐IP) Assay

To corroborate the interaction between DTX2 and Notch2, co‐immunoprecipitation (Co‐IP) assays were conducted in both exogenous and endogenous settings.

Co‐IP in 293 T cells: 293 T cells were subjected to transfection with a Myc‐tagged Notch2 overexpression plasmid, employing Lipofectamine 3000 (Thermo Fisher Scientific, Waltham, MA, USA). Subsequent to a 48‐h period, cell lysates were prepared in a lysis buffer containing protease inhibitors. Subsequently, the lysates were incubated with either IgG control antibody or anti‐Myc antibody at 4°C for a period of 12 h. Subsequently, Protein A/G magnetic beads were added to capture the antibody‐protein complexes. Subsequent to the washing step, the immunoprecipitated proteins were eluted and subjected to analysis by Western blot, utilizing antibodies directed against DTX2 and Myc‐Notch2. The presence of DTX2 in the Myc‐Notch2 immunoprecipitates indicated a specific interaction between the two proteins.

Co‐IP in SH‐SY5Y cells: To conduct endogenous protein interaction studies, SH‐SY5Y cells were lysed in a buffer containing protease inhibitors. Subsequently, the lysates were subjected to incubation with either IgG control antibody, anti‐DTX2 antibody, or anti‐Notch2 antibody at 4°C for a period of 16 h. Protein A/G magnetic beads were utilized to capture the antibody‐protein complexes. Following extensive washing, the immunoprecipitated proteins were eluted and subjected to analysis by Western blot using antibodies against DTX2 and Notch2. The detection of DTX2 in the Notch2 immunoprecipitates confirmed the endogenous interaction between DTX2 and Notch2.

### 2.24 Assessment of DTX2‐Mediated Notch2 Ubiquitination and Protein Stability

A rigorous investigation was conducted to ascertain the effect of DTX2 on Notch2 ubiquitination in SH‐SY5Y cells. To this end, cells were subjected to transfection with a DTX2 overexpressing plasmid and HA‐tagged ubiquitin (HA‐Ub) using Lipofectamine 3000 (Thermo Fisher Scientific, Waltham, MA, USA). Following a 24‐h period of incubation, the cells were treated with the proteasome inhibitor MG132 (20 μM) for a duration of 4 h. This treatment was found to stabilize ubiquitinated proteins. Cell lysates were prepared and analyzed by Western blot analysis using antibodies against Notch2, DTX2, and HA to assess the levels of ubiquitinated Notch2.

The present study sought to evaluate the impact of DTX2‐mediated Notch2 ubiquitination on protein stability. To this end, SH‐SY5Y cells were utilized as a model system and were subjected to transfection with the DTX2 overexpressing plasmid. Following a 24‐h period of incubation, the cells were treated with cycloheximide (CHX, 50 μg/mL) for various time points (0–10 h) to inhibit protein synthesis. Cell lysates were collected at predetermined time points and subsequently analyzed by Western blot analysis, utilizing antibodies directed against Notch2.

### 2.25 Western Blot Analysis of DTX2 and Its Mutants on Notch2 Protein Expression

293 T cells were transfected with HA‐tagged wild‐type (WT) DTX2, DTX2 mutants (△RING‐HA, △WWE1‐HA, △WWE2‐HA), and Notch2‐Flag. Western blotting was performed to detect Flag‐ and HA‐complex protein expression [22].

### 2.26 Statistical Analysis

The data are expressed as the mean ± standard deviation (SD). The data were tested for normality and found to follow a normal distribution (Shapiro–Wilk test, *p* > 0.05). To facilitate a comparison between two groups, the Student's *t*‐test was employed. For comparisons involving more than two groups, one‐way analysis of variance (ANOVA) was performed, followed by Bonferroni post hoc test for multiple comparisons. A *p*‐value less than 0.05 was considered statistically significant. All statistical analyses were conducted using GraphPad Prism 9 software and SPSS 26.0 (IBM, Armonk, NY, USA).

## 3 Results

### 3.1 Differentially Expressed Proteins in FOG Rat STR Tissues

**FIGURE 1:**
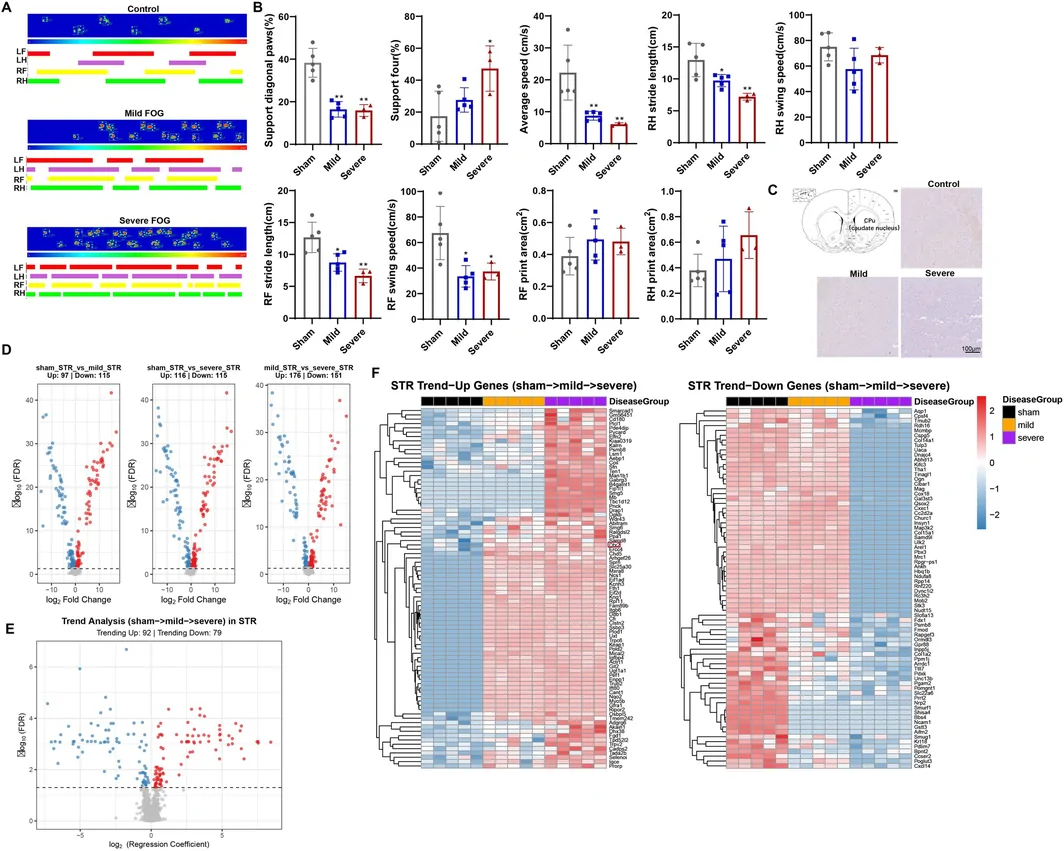
Differentially expressed proteins in FOG rat STR tissues. (A) FOG rat model was established by bilateral injections of 6‐OHDA into the dorsal striatum and bilateral infusions of immunotoxin 192 IgG‐saporin into the basal forebrain regions. Five weeks after the injection, each rat was subjected to three Catwalk runway tests. In the FOG group, rats were divided into the mild group (*n* = 8) and the severe group (*n* = 12) according to the test results. Five rats were randomly selected from each group and were tested using the Catwalk gait analysis. (B) A total of 5 rats from the sham group, 5 from the mild group, and 3 from the severe group finished the Catwalk gait analysis. Catwalk XT system was used to analyze the data. Statistical analysis: one‐way ANOVA followed by Bonferroni post hoc test; *p < 0.05, **p < 0.01 versus Sham. (C) Immunohistochemical staining was used to observe the number of dopaminergic neurons (tyrosine hydroxylase marked) in the CPu region of rats. Scale bar: 100 μm. (D) Proteomic analysis was conducted on the STR tissue samples from each group of rats. (E, F) To determine whether the abundance of proteins exhibits a unidirectional linear trend with the progression of FOG (from sham, mild, to severe), a linear regression analysis was conducted. CPu, caudate putamen; FDR, false discovery rate; FOG, freezing of gait; LF, left fore; LH, left hind; RF, right fore; RH, right hind; STR, striatum.

The FOG rat model was established by bilateral injections of 6‐OHDA into the dorsal striatum and bilateral infusions of immunotoxin 192 IgG‐saporin into the basal forebrain regions. Five weeks after the injection, each rat was subjected to three Catwalk runway tests. All rats in the sham group (*n* = 10) passed the tests. The probability of failing to complete the test due to severe episodes of FOG (initial hesitation or mid‐course falls) in the FOG group (*n* = 20) is 34 out of 60 tests (56.67%). In the FOG group, rats that successfully completed all three runway tests were classified as having mild impairments (*n* = 8), while the remaining rats were categorized as having severe impairments (*n* = 12). Notably, data from 10 rats was not obtained, and 2 rats had data collected once. A total of five rats were selected from each of the sham, mild, and severe groups for the purpose of testing using the Catwalk gait analysis. However, in the severe group, data from two rats could not be obtained. Consequently, a total of five rats from the sham group, five from the mild group, and three from the severe group were utilized for subsequent experiments. As illustrated in Figure 1A, the rats within each group exhibited characteristic gait patterns. In comparison with the sham group, the percentage of diagonal paw support was reduced in both mild and severe groups, while four paw support was increased in the severe group. Furthermore, the average speed, right hind (RH)/right fore (RF) stride length, and RF swing speed were also reduced in both mild and severe groups (Figure 1B). The employment of TH antibodies as a means of labeling dopaminergic neurons constituted a pivotal aspect of the study. The results of immunohistochemical staining demonstrated a decrease in the number of dopaminergic neurons in the CPu region of FOG rats (Figure 1C). These findings indicated the successful establishment of the FOG rat model.

Proteomic analysis was conducted on the STR tissue samples from each group of rats. A comparative analysis of the sequencing results from three groups of samples, arranged in pairs, has led to the identification of several proteins that have undergone either upregulation or downregulation (Figure 1D). In order to ascertain whether the abundance of proteins exhibits a unidirectional linear trend with the progression of FOG (from sham, mild, to severe), a linear regression analysis was conducted, which identified 92 upregulated proteins, including DTX2, and 79 downregulated proteins (Figure 1E,F). The absence of DTX2 has been demonstrated to enhance spinal cord regeneration and motor function recovery [23]. Therefore, it was hypothesized that DTX2 might exert a detrimental effect on the progression of FOG.

### 3.2 Interference With DTX2 Protects Human Dopamine‐Like Neural Cells From Neurotoxic Injury and Oxidative Stress

**FIGURE 2:**
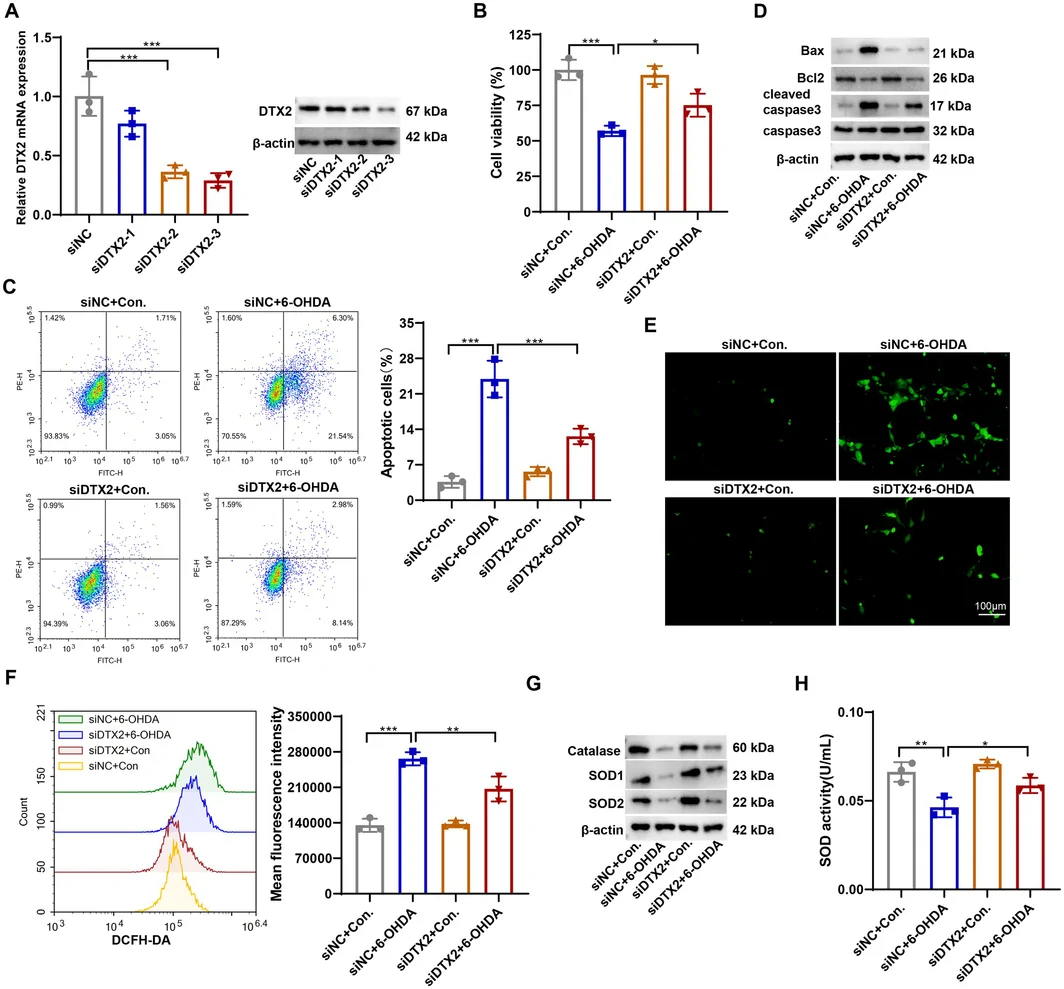
Interference with DTX2 protects human dopamine‐like neural cells from neurotoxic injury and oxidative stress. (A) Differentiated SH‐SY5Y cells were transfected with three different DTX2 interference sequences (siDTX2‐1/2/3). mRNA and protein levels of DTX2 were detected using qPCR and Western blot analysis. Statistical analysis: one‐way ANOVA followed by Bonferroni post hoc test; ***p < 0.001. *N* = 3. (B–G) After the transfection, differentiated SH‐SY5Y cells were treated with 6‐OHDA to induce a neurotoxic injury cell model. (B) Cell viability was detected using the MTT assay. (C) Cell apoptosis was detected using the Annexin V/PI apoptosis assay. (D) Expressions of apoptosis‐related proteins were detected using Western blot analysis. (E, F) Intracellular ROS levels were detected using DCFH‐DA staining. Relative fluorescence intensity was detected using flow cytometry. (G) Expressions of antioxidant proteins were detected using Western blot analysis. (H) Intracellular SOD levels were detected using a colorimetric SOD activity assay kit. Statistical analysis: One‐way ANOVA followed by Bonferroni post hoc test; *p < 0.05, **p < 0.01, ***p < 0.001. *N* = 3. 6‐OHDA, 6‐hydroxydopamine; Con, control; DCFH‐DA, 2′,7′‐dichlorofluorescein diacetate; NC, negative control; ROS, reactive oxygen species; SOD, superoxide dismutase.

The induction of human dopamine‐like neural cells was achieved through the utilization of SH‐SY5Y cells, which are human neuroblastoma cells. A notable absence of significant alterations in DTX2 expression was observed both prior to and following the differentiation of SH‐SY5Y cells (Figure S1). The differentiated SH‐SY5Y cells were then subjected to transfection with three distinct DTX2 interference sequences, designated as siDTX2‐1, siDTX2‐2, and siDTX2‐3, respectively. Following the verification of transfection efficiency (Figure 2A, in which siDTX2‐3 was selected), the subjects were subjected to 6‐OHDA treatment, a method that induces a neurotoxic injury cell model. The activity of the differentiated SH‐SY5Y cells was assessed by applying different gradient concentrations of 6‐OHDA. The investigation revealed a concentration‐dependent decline in the activity of differentiated SH‐SY5Y cells, with a decrease ranging from 7.5 to 15 μg/mL of 6‐OHDA. At a concentration of 10 μg/mL, the cell activity was found to be approximately 57.17% (Figure S2). Consequently, this concentration (10 μg/mL) was selected for subsequent experiments.

While interference with DTX2 did not result in a substantial impact on the activity of differentiated SH‐SY5Y cells (Figure 2B, column 1 vs. 3), interference with DTX2 did lead to a significant increase in cell activity of differentiated SH‐SY5Y cells under 6‐OHDA treatment conditions (Figure 2B, column 2 vs. 4). Interference with DTX2 significantly reduced cell apoptosis of differentiated SH‐SY5Y cells under 6‐OHDA treatment (Figure 2C, column 2 vs. 4) with corresponding trends of apoptosis‐related proteins (Figure 2D). The aforementioned results indicated that interference with DTX2 offers a degree of protection to SH‐SY5Y cells from the neurotoxic effects of 6‐OHDA.

As indicated in the relevant literature, oxidative stress has been demonstrated to play a pivotal role in the neuronal injury associated with PD [24, 25]. Consequently, the presence of ROS was detected in 6‐OHDA‐induced SH‐SY5Y cells. The results demonstrated that 6‐OHDA treatment led to a substantial increase in intracellular ROS levels in differentiated SH‐SY5Y cells. However, interference with DTX2 significantly reduced the intracellular ROS levels under 6‐OHDA treatment (Figure 2E,F). The present study employed Western blot analysis to detect antioxidant proteins, including SOD1, SOD2, and Catalase. The results demonstrated that 6‐OHDA treatment led to a decrease in the expression of SOD1, SOD2, and Catalase proteins in differentiated SH‐SY5Y cells. However, interference with DTX2 resulted in an increase in these expressions under 6‐OHDA treatment (Figure 2G). Furthermore, treatment with 6‐OHDA led to a decline in SOD activity in differentiated SH‐SY5Y cells. However, interference with DTX2 resulted in a significant increase in SOD activity under 6‐OHDA treatment conditions (Figure 2H). The aforementioned results indicated that DTX2 interference alleviates the oxidative stress induced by 6‐OHDA in SH‐SY5Y cells.

### 3.3 Interference With DTX2 Increases Dopamine and TH Expression in 6‐OHDA‐Treated Human Dopamine‐Like Neural Cells

**FIGURE 3:**
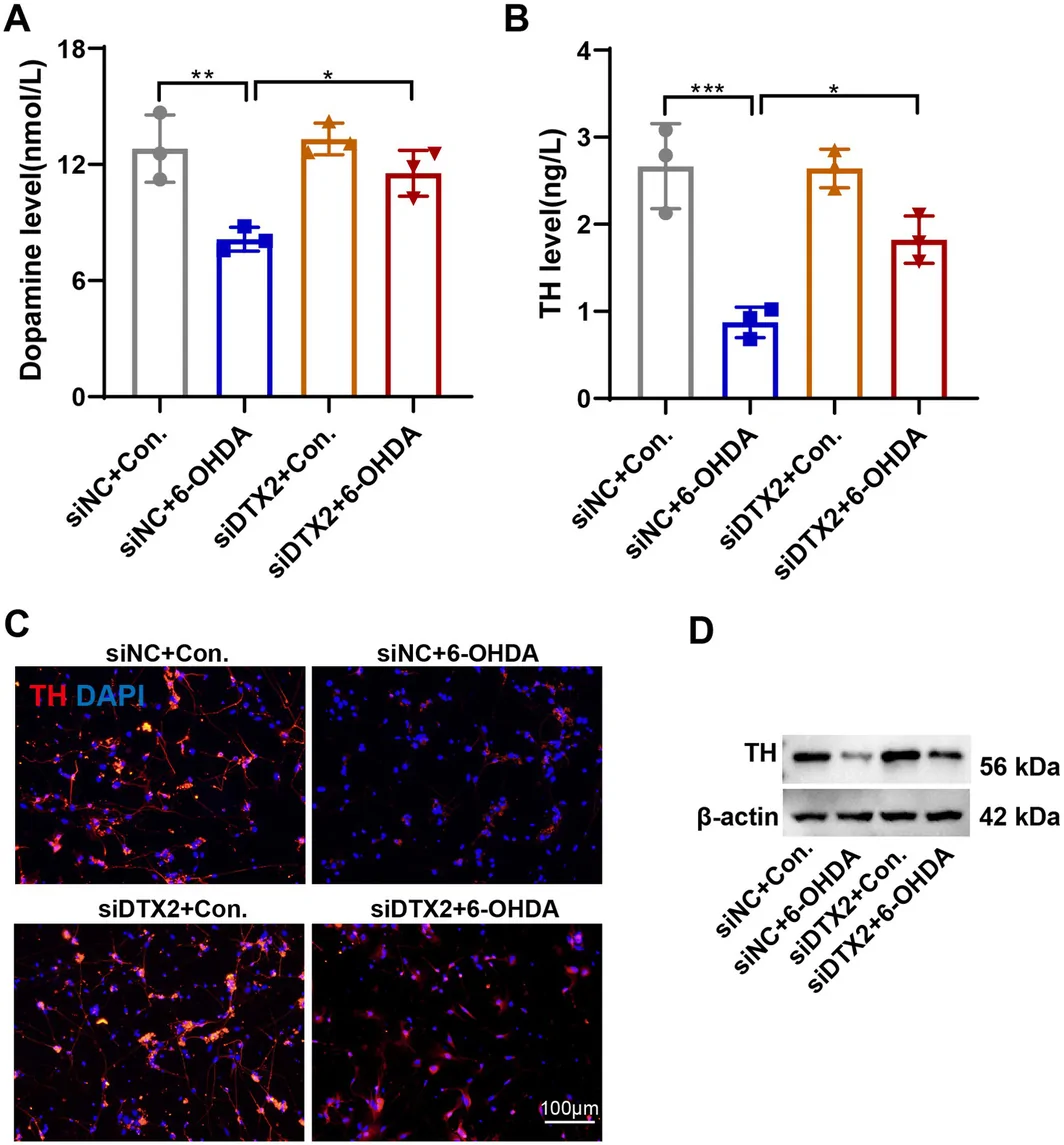
Interference with DTX2 increases dopamine and TH expression in 6‐OHDA‐treated human dopamine‐like neural cells. After the interference with DTX2, differentiated SH‐SY5Y cells were treated with 6‐OHDA to induce a neurotoxic injury cell model. (A) Dopamine levels in cell lysates were detected using the Dopamine ELISA Kit. (B) TH levels in cell lysates were detected using the Human Tyrosine Hydroxylase ELISA Kit. (C) Immunofluorescence staining of differentiated SH‐SY5Y cells using the TH antibody. (D) Expression of TH protein was detected using Western blot analysis. Statistical analysis: one‐way ANOVA followed by Bonferroni post hoc test; *p < 0.05, **p < 0.01, ***p < 0.001. *N* = 3. 6‐OHDA, 6‐hydroxydopamine; Con, control; ELISA, enzyme‐linked immunosorbent assay; NC, negative control; TH, tyrosine hydroxylase.

Since interference with DTX2 protects human dopamine‐like neural cells from neurotoxic injury and oxidative stress, we next investigated whether it increases dopamine and TH expression. The results showed that 6‐OHDA treatment reduces dopamine levels in differentiated SH‐SY5Y cells, and interference with DTX2 restored dopamine levels (Figure 3A). TH is the rate‐limiting enzyme for dopamine synthesis. 6‐OHDA treatment reduces TH levels in differentiated SH‐SY5Y cells, while interference with DTX2 restored TH levels (Figure 3B). Immunofluorescence staining and Western blot results also confirmed that interference with DTX2 significantly restored TH expression in differentiated SH‐SY5Y cells under 6‐OHDA treatment (Figure 3C,D). These data indicated that interference with DTX2 increases dopamine and TH expression in 6‐OHDA‐treated human dopamine‐like neural cells.

### 3.4 Striatal Interference With DTX2 Alleviates Gait Abnormalities in FOG Rats

**FIGURE 4:**
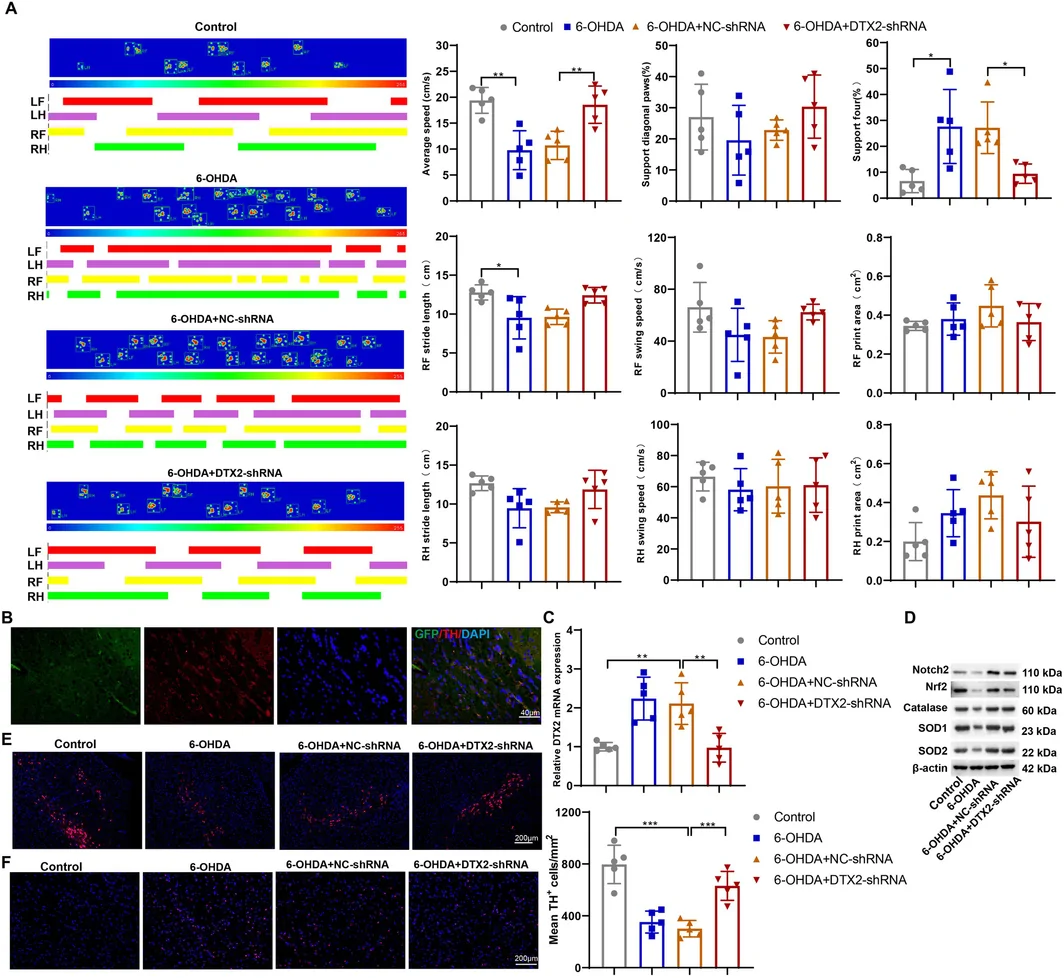
Striatal interference with DTX2 alleviates gait abnormalities in FOG rats. In FOG rats, 3 weeks after 6‐OHDA injection, AAV vectors carrying shRNAs against DTX2 were injected into STR tissues to interfere with DTX2 (*n* = 5 in each group). (A) Rats were subjected to Catwalk gait analysis. Catwalk XT system was used to analyze the data. (B) Immunofluorescence staining of STR tissues using the GFP and TH antibodies. (C) Expression of DTX2 mRNA in SNpc was detected using qPCR. (D) The expression of Notch2, Nrf2, and antioxidant proteins in the substantia nigra pars compacta of rats was detected using Western blotting. (E) Immunofluorescence staining of SNpc using the TH antibody. The mean TH positive cells were quantified in the right lower panel. (F) Cell apoptosis in SNpc was detected using the TUNEL assay. Statistical analysis: one‐way ANOVA followed by Bonferroni post hoc test; *p < 0.05, **p < 0.01, ***p < 0.001. 6‐OHDA, 6‐hydroxydopamine; AAV, adeno‐associated virus; FOG, freezing of gait; LF, left fore; LH, left hind; NC, negative control; RF, right fore; RH, right hind; shRNA, short hairpin RNA; SNpc, substantia nigra pars compacta; STR, striatum; TH, tyrosine hydroxylase; TUNEL, terminal deoxynucleotidyl transferase dUTP nick end labeling.

Interference with DTX2 was also performed in vivo. In FOG rats, 3 weeks after 6‐OHDA injection, AAV vectors carrying shRNAs against DTX2 were injected into STR tissues. After 2 weeks of AAV injection, the results of Catwalk gait analysis showed that DTX2‐shRNA alleviated gait abnormalities of FOG rats, markedly increasing average speed and decreasing four paw support (Figure 4A). AAV vector expression was detected in the STR tissues of rat brain (Figure 4B), and DTX2 mRNA expression was significantly reduced in SNpc (Figure 4C). Consistent with the findings at the cellular results, DTX2 knockdown increased the expression of Notch2, Nrf2, and antioxidant proteins in the substantia nigra pars compacta of FOG rats (Figure 4D). In addition, DTX2‐shRNA injection increased the number of dopaminergic neurons in SNpc of FOG rats (Figure 4E) with the decrease of TUNEL positive cells (Figure 4F). The above findings indicated that striatal interference with DTX2 alleviates gait abnormalities in FOG rats.

### 3.5 DTX2 Induces Oxidative Stress, Resulting in Neurotoxic Injury to Human Dopamine‐Like Neural Cells

**FIGURE 5:**
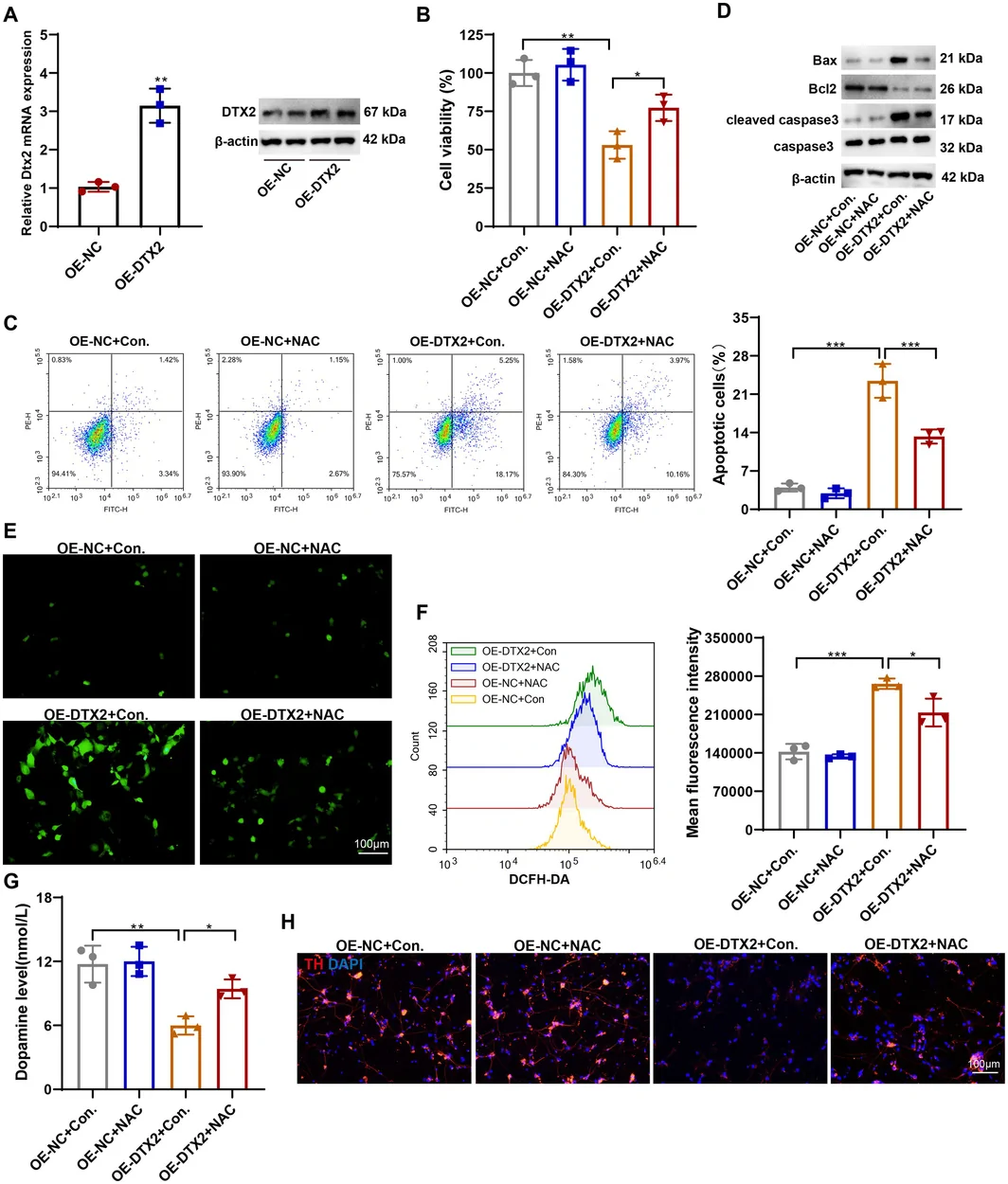
DTX2 induces oxidative stress, resulting in neurotoxic injury to human dopamine‐like neural cells. (A) DTX2 was overexpressed in differentiated SH‐SY5Y cells using plasmids. mRNA and protein levels of DTX2 before and after the transfection were detected using qPCR and Western blot analysis. Statistical analysis: unpaired Student's *t*‐test. **p < 0.01 versus OE‐NC. *N* = 3. (B–H) After overexpressing DTX2, differentiated SH‐SY5Y cells were treated with the ROS inhibitor NAC. (B) Cell viability was detected using the MTT assay. (C) Cell apoptosis was detected using the Annexin V/PI apoptosis assay. (D) Expressions of apoptosis‐related proteins were detected using Western blot analysis. (E, F) Intracellular ROS levels were detected using DCFH‐DA staining. Relative fluorescence intensity was detected using flow cytometry. (G) Dopamine levels in cell lysates were detected using the Dopamine ELISA Kit. (H) Immunofluorescence staining of differentiated SH‐SY5Y cells using the TH antibody. Statistical analysis: one‐way ANOVA followed by Bonferroni post hoc test; *p < 0.05, **p < 0.01, ***p < 0.001. *N* = 3. 6‐OHDA, 6‐hydroxydopamine; Con, control; DCFH‐DA, 2′,7′‐dichlorofluorescein diacetate; ELISA, enzyme‐linked immunosorbent assay; NC, negative control; OE, overexpression; ROS, reactive oxygen species; TH, tyrosine hydroxylase.

After overexpressing DTX2 in differentiated SH‐SY5Y cells, the transfection efficiency was verified (Figure 5A), and then treated with the ROS inhibitor NAC. Overexpression of DTX2 reduced the cell viability of differentiated SH‐SY5Y cells, while NAC restored cell viability (Figure 5B). Overexpression of DTX2 significantly increased cell apoptosis of differentiated SH‐SY5Y cells, while NAC treatment reduced cell apoptosis (Figure 5C) with corresponding trends of apoptosis‐related proteins (Figure 5D). In addition, overexpression of DTX2 significantly increased the intracellular ROS levels in differentiated SH‐SY5Y cells, while NAC reduced the intracellular ROS levels (Figure 5E,F). Overexpression of DTX2 reduces dopamine levels in differentiated SH‐SY5Y cells, and NAC restored dopamine levels (Figure 5G). Meanwhile, overexpression of DTX2 reduced TH expression in differentiated SH‐SY5Y cells, while NAC restored the TH expression (Figure 5H). These data indicate that overexpression of DTX2 induces oxidative stress, resulting in neurotoxic injury to human dopamine‐like neural cells.

### 3.6 DTX2 Induces Injury to Human Dopamine‐Like Neural Cells by Reducing Notch2 Protein Levels

**FIGURE 6:**
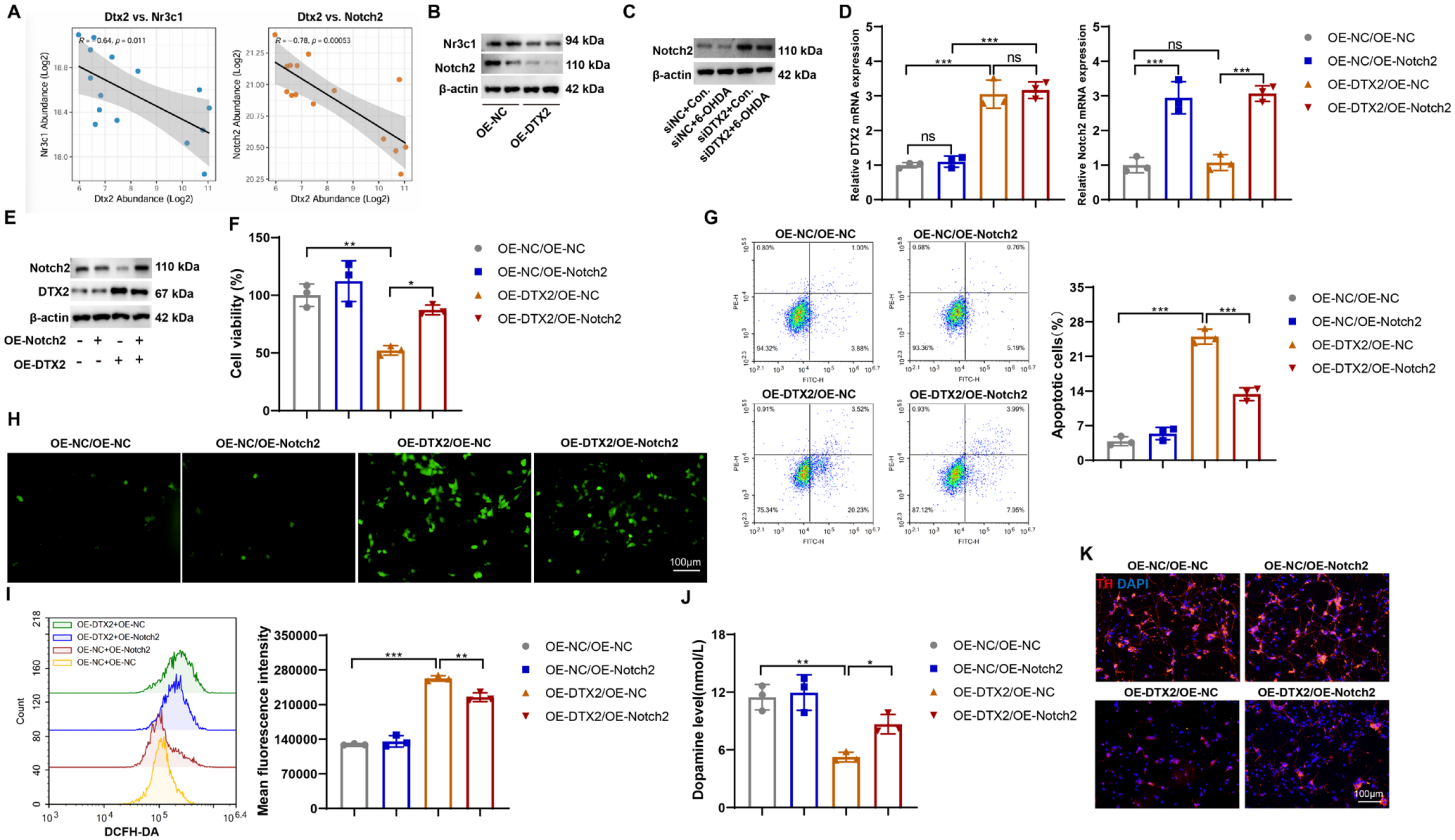
DTX2 induces injury to human dopamine‐like neural cells by reducing Notch2 protein levels. (A) According to proteomic sequencing results and Ubibrowser 2.0 results, Nr3c1 and Notch2 were identified as potential substrate proteins of DTX2, whose expression levels in STR tissues showed significant negative correlation with DTX2 expression. (B) Differentiated SH‐SY5Y cells were transfected with DTX2 overexpression plasmids. Expressions of Nr3c1 and Notch2 proteins were detected using Western blot analysis. (C) After the interference with DTX2, differentiated SH‐SY5Y cells were treated with 6‐OHDA to induce a neurotoxic injury cell model. Expression of Notch2 protein was detected using Western blot analysis. (D–K) Differentiated SH‐SY5Y cells were co‐transfected with plasmids (DTX2 and Notch2 overexpression plasmids). (D) DTX2 and Notch2 mRNA expression levels were measured using qPCR. (E) Expression of Notch2 and DTX2 proteins was detected using Western blot analysis. (F) Cell viability was detected using the MTT assay. (G) Cell apoptosis was detected using the Annexin V/PI apoptosis assay. (H, I) Intracellular ROS levels were detected using DCFH‐DA staining. Relative fluorescence intensity was detected using flow cytometry. (J) Dopamine levels in cell lysates were detected using the Dopamine ELISA Kit. (K) Immunofluorescence staining of differentiated SH‐SY5Y cells using the TH antibody. Statistical analysis: one‐way ANOVA followed by Bonferroni post hoc test; *p < 0.05, **p < 0.01, ***p < 0.001. *N* = 3. 6‐OHDA, 6‐hydroxydopamine; Con, control; DCFH‐DA, 2′,7′‐dichlorofluorescein diacetate; ELISA, enzyme‐linked immunosorbent assay; NC, negative control; OE, overexpression; ROS, reactive oxygen species; STR, striatum; TH, tyrosine hydroxylase.

Given that DTX2 is an E3 ubiquitin ligase, its primary mechanism of action involves regulating the degradation and function of various substrate proteins through ubiquitin modification, thereby influencing cellular biological behavior [26]. Therefore, sequencing samples from STR tissues were first isolated based on the proteome expression matrix. Pearson correlation analysis between DTX2 and other proteins revealed 416 proteins exhibiting a significant negative correlation with DTX2 expression (Table S1). Subsequently, using Ubibrowser 2.0 (a database predicting ubiquitin‐related interactions), DTX2 was selected as an “E3 ligase” to predict potential substrate proteins it could bind as an E3 ubiquitin ligase. This yielded 39 candidate proteins (Table S2). Integrating these two findings, we identified two DTX2 potential substrate proteins, Nr3c1 and Notch2, whose expression levels in STR tissues showed significant negative correlation with DTX2 expression (Figure 6A).

In the in vitro experiments, after overexpressing DTX2 in differentiated SH‐SY5Y cells, Notch2 protein expression is significantly reduced, but there is no obvious effect on the expression level of Nr3c1 protein (Figure 6B). Further experiments revealed that Notch2 expression was downregulated in 6‐OHDA‐treated differentiated SH‐SY5Y cells, whereas interference with DTX2 significantly increased Notch2 expression under 6‐OHDA treatment. This suggests that Notch2 may serve as a downstream target molecule of DTX2 (Figure 6C). Although the specific role of Notch2 protein in dopaminergic neuron loss in PD remains unclear, studies indicate that Notch2‐associated receptor 2 knockout mice exhibit PD‐like symptoms accompanied by substantia nigra dopaminergic neuron loss and increased α‐synuclein levels [27]. Therefore, we hypothesized that Notch2 may exert regulatory effects on dopaminergic neuron survival and function. To validate this hypothesis, differentiated SH‐SY5Y cells were transfected with a Notch2 interference sequence. After confirming transfection efficiency (Figure S3A), we found that Notch2 interference significantly reduced the activity of differentiated SH‐SY5Y cells (Figure S3B), promoted apoptosis (Figure S3C), and increased intracellular ROS levels (Figure S3D,E). Furthermore, Notch2 interference decreased TH expression in differentiated SH‐SY5Y cells (Figure S3F). These results indicated that Notch2 plays a crucial regulatory role in both the activity and TH expression of human dopamine‐like neural cells.

To validate the post‐translational regulation of Notch2 by DTX2, we examined both mRNA and protein levels. The results demonstrated that DTX2 and Notch2 overexpression did not affect each other's mRNA levels (Figure 6D), whereas DTX2 overexpression significantly decreased Notch2 protein levels (Figure 6E). Functional rescue experiments confirmed that Notch2 overexpression reversed DTX2‐induced reductions in cell viability, increased apoptosis, ROS accumulation, decreased dopamine, and downregulated TH expression (Figure 6F–K), indicating that DTX2 mediates neurotoxicity through reducing Notch2 protein levels.

### 3.7 Notch2 Promotes Nrf2 Transcription, Helping to Maintain the Antioxidant Capacity of Human Dopamine‐Like Neural Cells

**FIGURE 7:**
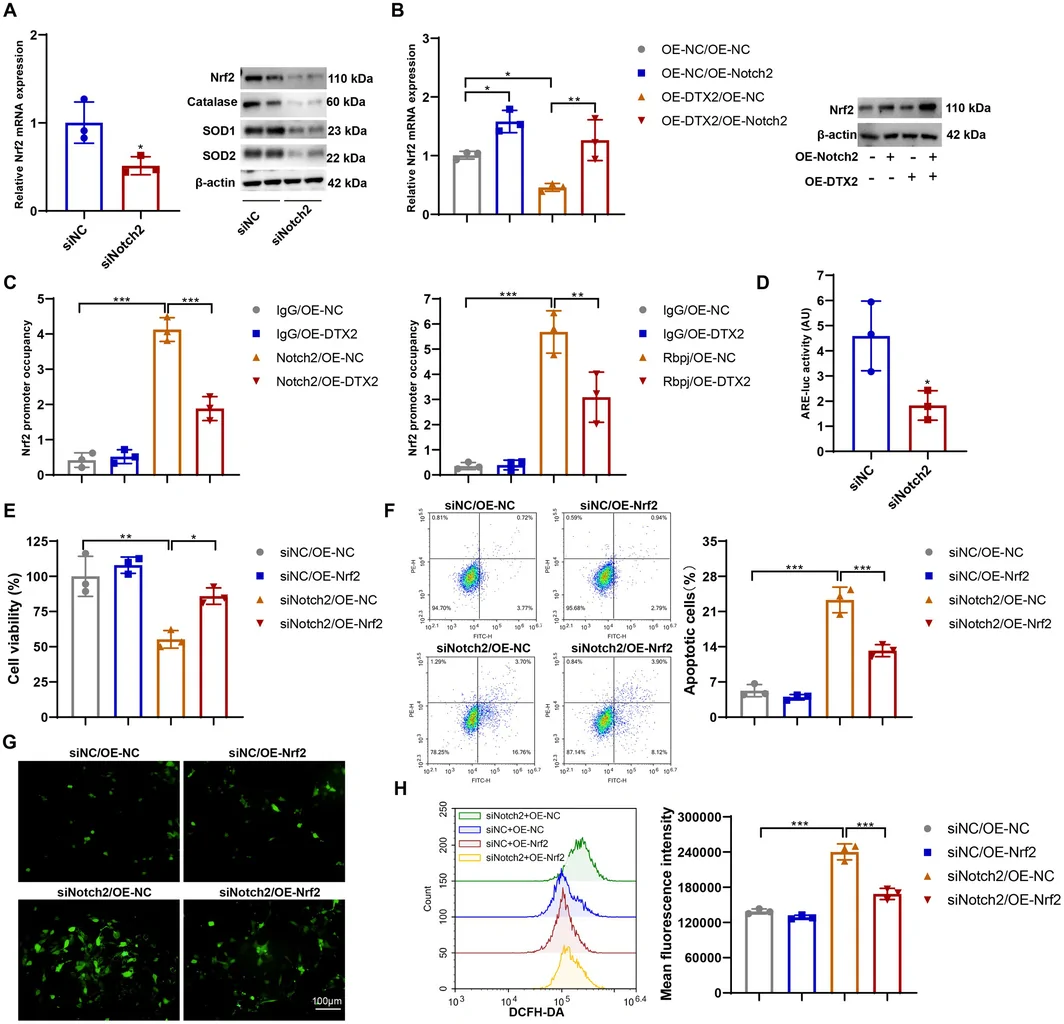
Notch2 promotes Nrf2 transcription, helping to maintain the antioxidant capacity of human dopamine‐like neural cells. (A) Differentiated SH‐SY5Y cells were transfected with a Notch2 interference sequence. Nrf2 mRNA expression was detected using qPCR. Expressions of Nrf2 and antioxidant proteins were detected using Western blot analysis. Statistical analysis: unpaired Student's *t*‐test. *p < 0.05 versus siNC. *N* = 3. (B) Differentiated SH‐SY5Y cells were co‐transfected with plasmids (DTX2 and Notch2 overexpression plasmids). mRNA and protein levels of Nrf2 were detected using qPCR and Western blot analysis. Statistical analysis: One‐way ANOVA followed by Bonferroni post hoc test; *p < 0.05, **p < 0.01. *N* = 3. (C) Differentiated SH‐SY5Y cells were transfected with DTX2 overexpression plasmids. ChIP was performed to investigate the binding of Notch2 and Rbpj to the Nrf2 promoter region. Statistical analysis: one‐way ANOVA followed by Bonferroni post hoc test; **p < 0.01, ***p < 0.001. *N* = 3. (D) Differentiated SH‐SY5Y cells were transfected with a Notch2 interference sequence. A dual‐luciferase reporter assay was employed to evaluate the transcriptional activity of Nrf2. Statistical analysis: unpaired Student's *t*‐test. *p < 0.05 versus siNC. *N* = 3. (E–H) Differentiated SH‐SY5Y cells were co‐transfected with Notch2 interference sequences and Nrf2 overexpression plasmids. (E) Cell viability was detected using the MTT assay. (F) Cell apoptosis was detected using the Annexin V/PI apoptosis assay. (G, H) Intracellular ROS levels were detected using DCFH‐DA staining. Relative fluorescence intensity was detected using flow cytometry. Statistical analysis: one‐way ANOVA followed by Bonferroni post hoc test; *p < 0.05, **p < 0.01, ***p < 0.001. *N* = 3. 6‐OHDA, 6‐hydroxydopamine; ChIP, Chromatin immunoprecipitation; DCFH‐DA, 2′,7′‐dichlorofluorescein diacetate; NC, negative control; OE, overexpression; ROS, reactive oxygen species.

Following Notch2 activation, its cleavage products enter the nucleus and bind to the transcription factor recombination signal binding protein for immunoglobulin kappa J region (Rbpj) to form a transcription activation complex, thereby regulating the expression of downstream genes [28]. Previous studies have demonstrated the presence of a highly conserved Rbpj binding site within the Nrf2 promoter region, and that Notch signaling pathway activation increases Nrf2 transcription [29]. To validate Notch2 transcriptional regulation of Nrf2 in human dopamine‐like neural cells, differentiated SH‐SY5Y cells were transfected with a Notch2 interference sequence. Following Notch2 interference, Nrf2 mRNA and protein expression were significantly reduced, and the expression of ROS‐scavenging proteins (SOD1, SOD2, and Catalase) was decreased (Figure 7A). DTX2 overexpression also suppressed Nrf2 expression, whereas Notch2 overexpression reversed this effect (Figure 7B). ChIP assays showed that the Notch2‐Rbpj complex was enriched at the Nrf2 promoter, and DTX2 overexpression attenuated this enrichment (Figure 7C); dual‐luciferase reporter assays confirmed that Notch2 knockdown inhibited Nrf2 transcriptional activity (Figure 7D). Functional experiments demonstrated that Nrf2 overexpression reversed cellular damage induced by Notch2 knockdown (Figure 7E–H).

### 3.8 DTX2 Mediates the Ubiquitination of Notch2, Thereby Promoting Its Degradation

**FIGURE 8:**
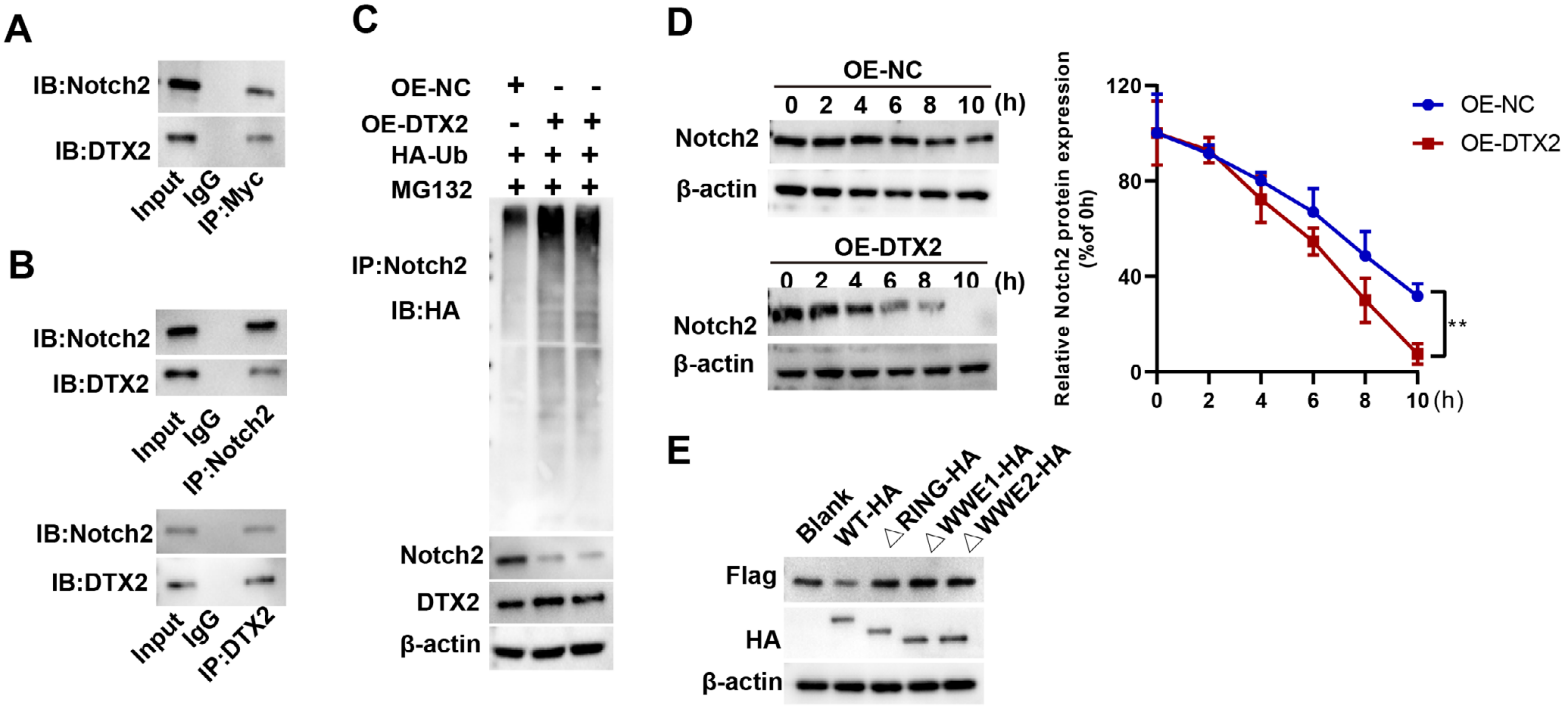
DTX2 mediates the ubiquitination of Notch2, thereby promoting its degradation. To confirm the interaction between DTX2 and Notch2, Co‐IP assays were performed in both exogenous (A) and endogenous (B) settings. (C) SH‐SY5Y cells were transfected with a DTX2 overexpression plasmid and HA‐Ub. After 24 h, cells were treated with the proteasome inhibitor MG132 (20 μM) for 4 h to stabilize ubiquitinated proteins. Cell lysates were prepared and analyzed by Western blot analysis using antibodies against Notch2 and HA‐Ub to assess the levels of ubiquitinated Notch2. (D) SH‐SY5Y cells were transfected with the DTX2 overexpression plasmid. After 24 h, cells were treated with CHX (50 μg/mL) for various time points (0–10 h) to inhibit protein synthesis. Cell lysates were collected at each time point and analyzed by Western blot analysis using antibodies against Notch2. (E) Two hundred and ninety‐three T cells were co‐transfected with Notch2‐Flag and HA‐tagged wild‐type (WT) DTX2 or DTX2 mutants (△RING‐HA, △WWE1‐HA, △WWE2‐HA). Western blot analysis showed that wild‐type DTX2 decreased Notch2 protein levels, whereas DTX2 mutants lacking the RING domain or WWE domains failed to reduce Notch2 expression. Statistical analysis: unpaired Student's *t*‐test. **p < 0.01. *N* = 3. CHX, cycloheximide; Co‐IP, co‐immunoprecipitation; HA‐Ub, HA‐tagged ubiquitin; IB, immunoblotting; IP, immunoprecipitation; NC, negative control; OE, overexpression.

Next, we further elucidated the mechanism by which DTX2 regulates Notch2 expression. Co‐IP experiments confirmed the existence of endogenous and exogenous interactions between DTX2 and Notch2 (Figure 8A,B). DTX2 overexpression promoted Notch2 ubiquitination (Figure 8C) and accelerated its protein degradation (Figure 8D). Structure–function analysis revealed that wild‐type DTX2 reduced Notch2 protein levels, whereas DTX2 mutants lacking the RING domain or WWE domains showed no such effect (Figure 8E), confirming that DTX2 mediates Notch2 ubiquitination and degradation through its E3 ligase activity.

## 4 Discussion

This study delineates a DTX2‐driven mechanism of dopaminergic neurotoxicity that fuels freezing‐of‐gait (FOG) progression. Proteomic screening of FOG rat striatum showed DTX2 up‐regulation that scaled with phenotype severity. In 6‐OHDA‐challenged, dopaminergic‐like SH‐SY5Y cells, siRNA‐mediated DTX2 knockdown reversed oxidative injury: viability rose, apoptosis and ROS fell, and dopamine together with tyrosine hydroxylase were restored to control levels. By coupling these in vitro gains to the in vivo correlation data, DTX2 emerges as a druggable node whose inhibition could slow or prevent FOG in Parkinson's disease.

E3 ubiquitin ligases have been demonstrated to fulfill a pivotal function in the regulation of cellular homeostasis, particularly in the context of neurodegenerative diseases. In these diseases, E3 ligases have been observed to modulate protein degradation, recognize misfolded proteins, and influence inflammatory responses [30]. DTX2 belongs to the E3 ubiquitin ligase family of proteins, which are involved in the process of protein ubiquitination, the process by which ubiquitin molecules are attached to target proteins [31]. Ubiquitination, a pivotal post‐translational modification, plays a critical role in regulating protein stability, localization, and interactions [32]. Through ubiquitination, DTX2 can mark target proteins for proteasomal degradation or alter their cellular functions [33]. The C‐terminal domain of DTX2 has been demonstrated to recognize and recruit ADP‐ribosylated proteins for ubiquitination [34]. However, research on the specific role of DTX2 in neurodegenerative diseases remains relatively scarce. This study is pioneering in its exploration of the role of DTX2 in PD‐associated FOG progression and the underlying molecular mechanisms.

Oxidative stress arises when cellular oxidants outnumber antioxidants, leading to damage and dysfunction. Among the regulators of this imbalance, E3 ubiquitin ligases are key: the ligase HACE1, for example, curbs neuroinflammation in Parkinson models by ubiquitinating and degrading the small GTPase Rac1, a driver of oxidative injury [35]. Another E3 ubiquitin ligase, Parkin, has been demonstrated to fulfill a regulatory function in mitochondrial autophagy. Moreover, mitochondrial dysfunction has been demonstrated to be closely associated with oxidative stress [36]. However, the role of DTX2 in the oxidative stress of dopaminergic neurons remains to be elucidated. The present study found that interfering with DTX2 expression protects human dopaminergic neurons from oxidative stress, with the mechanism potentially involving DTX2‐mediated ubiquitination and degradation of Notch2.

DTX2 occupies a distinctive niche among PD‐relevant E3 ubiquitin ligases. Unlike Parkin—which safeguards mitochondrial quality through mitophagy and whose mutations cause familial early‐onset PD—DTX2 regulates oxidative stress tolerance via Notch2 ubiquitination and Nrf2 transcriptional control, independent of mitochondrial surveillance. Similarly, HACE1 mitigates neuroinflammation through Rac1 degradation and cytoskeletal signaling, whereas DTX2 directly controls transcriptional antioxidant programs [37]. This functional divergence renders DTX2 particularly attractive for sporadic PD with FOG: Parkin‐targeted strategies remain limited to rare genetic forms, while DTX2 upregulation appears as an acquired, disease‐progression‐associated event, suggesting that DTX2 inhibition could halt or reverse FOG in established PD rather than merely preventing early neurodegeneration.

The Notch signaling pathway plays a complex and multifaceted role in neurodegenerative diseases, possessing both the potential to promote neuroprotection and the capacity to exacerbate disease progression [38]. The Notch signaling pathway is a highly conserved intercellular communication mechanism activated through interactions between Notch receptors and their ligands, such as Delta and Serrate/Jagged [39]. Upon receptor activation, the intracellular domain (NICD) is released and transported to the nucleus, where it binds to transcription factors to regulate the expression of downstream target genes, thereby influencing cell fate, proliferation, and differentiation [40]. In PD, the Notch signaling pathway has been associated with PD‐related lysosomal dysfunction [41]. Additionally, the Notch signaling pathway also influences the aggregation of α‐synuclein [42]. This study is the first to implicate Notch signaling in freezing‐of‐gait progression. Activated Notch2 assembles a transcriptional activator with Rbpj that drives Nrf2 expression and sustains neuronal antioxidant capacity; conversely, Notch2 knockdown lowers Nrf2 and its downstream antioxidant enzymes, amplifying oxidative stress. Co‐transfection and ChIP experiments revealed that DTX2 blunts this protective axis by ubiquitinating and depleting Notch2, thereby suppressing Nrf2 transcription and crippling the antioxidant defense of human dopaminergic‐like neurons. Additionally, the Keap1/Nrf2 signaling pathway serves as one of the major neuroprotective mechanisms and acts as a critical regulator of antioxidant, anti‐inflammatory, and mitochondrial functions under stress conditions, playing an important role in the damage process of dopaminergic neurons in PD [43, 44]. However, our results showed that neither Notch2 overexpression nor DTX2 overexpression, alone or in combination, had a significant effect on KEAP1 expression, indicating that DTX2/Notch2‐mediated regulation of Nrf2 is Keap1‐independent (data not shown).

Our findings identify DTX2 as a tractable therapeutic node for FOG in PD, yet target specificity, potential adverse effects, and delivery strategies require careful consideration. DTX2 shares conserved RING and WWE domains with DTX1, DTX3, and DTX3L, raising off‐target concerns for catalytic inhibitors; selective targeting of DTX2‐Notch2 interfaces may improve specificity [45]. Systemic DTX2 inhibition risks disrupting physiological functions in neural plasticity, immune regulation, and ferroptosis resistance, necessitating localized delivery approaches. Given the absence of clinical DTX2 inhibitors and limited structural data, viable strategies include striatum‐specific AAV‐shRNA delivery (as demonstrated here), PROTAC‐mediated degradation, or allosteric modulators disrupting DTX2‐Notch2 binding [46]. Thus, circuit‐specific suppression within the nigrostriatal pathway—rather than global systemic inhibition—represents the most promising near‐term therapeutic approach.

The study has inherent constraints. Although the 6‐OHDA‐based FOG rat model reproduces key motor deficits seen in Parkinson's disease, it cannot mimic the full spectrum of human pathology—particularly the cognitive, affective, and autonomic disturbances characteristic of PD. Moreover, the lesion selectively targets dopaminergic neurons, whereas clinical parkinsonism involves additional neurotransmitter systems, including cholinergic and glutamatergic circuits [47]. Additionally, only male rats were used in this study because PD shows a higher incidence in men [13, 14]. Age‐matched male and female SD rats differ markedly in body weight, which influences behavioral readouts; consequently, we followed published precedent and selected males [15, 16]. This restriction may, however, limit the extrapolation of the findings to females. However, estrogen exerts bidirectional control over Notch signaling and Nrf2‐dependent antioxidant defenses, suggesting that DTX2 interference efficacy may differ between sexes. Additionally, the 6‐OHDA‐based FOG model selectively targets dopaminergic neurons without replicating the full spectrum of human PD pathology—including cognitive, affective, and autonomic disturbances, as well as cholinergic and glutamatergic circuit involvement. Future studies must validate this therapeutic strategy in female and ovariectomized models to determine whether sex modulates responses along the DTX2/Notch2/Nrf2 axis and extend findings to more comprehensive PD models.

In conclusion, under basal conditions Notch2 assembles a transcriptional activator that drives Nrf2 expression and preserves the antioxidant capacity of human dopaminergic‐like neurons. Pathologically, up‐regulated DTX2 ubiquitinates and degrades Notch2, attenuating Nrf2 transcription, amplifying oxidative stress, impairing neuronal viability, and thereby accelerating freezing‐of‐gait progression.

## Author Contributions

Q.Z. and L.Y. put forward the concept of the study, designed the study, and prepared the manuscript. X.Z. prepared the manuscript and contributed to the statistical analysis. J.Z. and L.L. contributed to the data acquisition. Y.C. and H.W. contributed to the quality control of data and algorithms. Z.L. edited the manuscript. All authors read and approved the final manuscript.

## Funding

This study was supported by the National Natural Science Foundation of China (No. 82171472), Joint Funds for Innovation of Science and Technology, Fujian Province (No. 2024Y9385), and Special Fund of Fujian Provincial Department of Finance (BPB‐2023YLH).

## Ethics Statement

The study was approved by the Research Medical Ethics Committee of the First Affiliated Hospital, Fujian Medical University (No. [2021]067). All animal experiments were carried out in accordance with the ARRIVE guidelines and the U.S. Public Health Service Policy on Humane Care and Use of Laboratory Animals.

## Conflicts of Interest

The authors declare no conflicts of interest.

## Supporting information


**Figure S1:** SH‐SY5Y cells (human neuroblastoma cells) were used to induce to human dopamine‐like neural cells. mRNA and protein levels of DTX2 before and after the transfection were detected using qPCR and Western blot analysis. Statistical analysis: unpaired Student's *t*‐test. *N* = 3.

**Figure S2:** By applying different gradient concentrations of 6‐OHDA, the activity of the differentiated SH‐SY5Y cells was assessed using the MTT assay. *N* = 3. 6‐OHDA, 6‐hydroxydopamine.

**Figure S3:** Differentiated SH‐SY5Y cells were transfected with a Notch2 interference sequence. (A) mRNA and protein levels of Notch2 before and after the transfection were detected using qPCR and Western blot analysis. (B) Cell viability was detected using the MTT assay. (C) Cell apoptosis was detected using the Annexin V/PI apoptosis assay. (D, E) Intracellular ROS levels were detected using DCFH‐DA staining. Relative fluorescence intensity was detected using flow cytometry. (F) Immunofluorescence staining of differentiated SH‐SY5Y cells using the TH antibody. Statistical analysis: unpaired Student's *t*‐test. **p < 0.01, ***p < 0.001 versus siNC. *N* = 3. DCFH‐DA, 2′,7′‐dichlorofluorescein diacetate; NC, negative control; ROS, reactive oxygen species; TH, tyrosine hydroxylase.

**Table S1:** Pearson correlation analysis between DTX2 and other proteins.

**Table S2:** The prediction of potential substrate proteins of DTX2.

## Data Availability

The datasets used and/or analyzed during the current study are available from the corresponding author on reasonable request.
